# Impact of emotional intelligence in resolving clinical conflicts among postgraduate residents of surgery

**DOI:** 10.12669/pjms.40.3.7363

**Published:** 2024

**Authors:** Syed Faraz Ul Hassan Shah Gillani, Rehan Ahmed Khan, Ahsan Sethi

**Affiliations:** 1 Syed Faraz Ul Hassan Shah Gillani, MS, MME Department of Orthopedic Surgery, King Edward Medical University/ Mayo Hospital,Lahore, Pakistan; 2 Rehan Ahmed Khan, FCPS, FRCS, MHPE, PhD Department of Medical education, Ripah International University, Islamabad, Pakistan; 3 Ahsan Sethi, BDS, MPH, MMEd, PhD Department of Public Health, College of Health Sciences, QU Health, Qatar University, Doha, Qatar

**Keywords:** Emotional intelligence, Postgraduate residents, Clinical conflict, Conflict resolution and surgery

## Abstract

**Background & Objective:**

Emotional intelligence (EI) can become a vital tool for resolving clinical conflicts (CC) in surgery. The postgraduate residents focus on the technical skills and undermine the soft skills required for their better training. Our aim was to determine the EI of postgraduate resident (PGR) years one & two in General and Orthopedic Surgery. The CC in their workplace and how they use their EI to resolve these conflicts.

**Methods:**

This mixed-method study was conducted from March 10, 2019 to May 28, 2020 at Departments of General and Orthopedic Surgery, Mayo Hospital, Lahore. The study was conducted in two phases 1 & 2. In Phase-1, one hundred PGR years one & two were administered the Mayor-Salovey-Caruso Emotional Intelligence test (MSCEIT) to measure EI. In phase-2, semi-structured in-depth interviews of 10 PGRs five with high and five with low EI were conducted to determine the CC and use of EI to resolve the CC at the workplace. A thematic analysis was done.

**Results:**

Out of 100 PGR, the mean EI score was 46.25±14.8 with a maximum score of 75.4, and a minimum score of 18.16 (p-value =0.775). Ninety-one (91%) have not improving EI, and 09 (09%) have considered developing EI. Five themes in four settings, including emergency, ward, elective operation theatre, and outpatient department (OPD) were determined. The emerged themes for the CC were nepotism, gender biases, burnout, lack of professionalism, and toxic culture. The following were CC management strategies: self-study, deceit, gender affinity, performing attention-attaining work, aggrieved reaction and being disgruntled when alone.

**Conclusion:**

None of the PGR was emotionally intelligent in overall grades, as well as a particular aspect of MSCEIT.

## INTRODUCTION

In the clinical setting, the ability to make clinical reasons and decisions in a demanding and emotionally charged situation is essential in the medical profession.^1^ Residents in surgery work as a team and coordinate the duties to achieve the learning outcomes of the training.^2^ They must provide safe and appropriate clinical care that requires the ability to do clinical reasoning and make the decision,^3^ Consequently, those people are preferred who do as they are told, out of a feeling of duty and obligation. The workplace environment is getting more competitive and challenging. It is considered a fundamental issue in the healthcare environment throughout the globe.^4^

Recently attention has been paid to EI in decision-making for safe and better clinical care. Differences and disagreement between leaders and residents may obscure the orientation and residents’ perspectives.^5^ Highly intelligent quotation (IQ) was considered as success in personal and professional life. Currently, EI requires proper management and organization in personal life and professional career.^6^-^8^ The emotional intelligence of doctor was found low in a local study.^9^ Individuals with good EI perform better in the workplace.^10^ The impulsive behaviour and frustration under challenging times of work also affect the team and their practices.^11^-^13^

In everyday routine in ward, the PGR encountered different challenges of inadequate infrastructure, lack of security, improper training standards, and extended duty hours. Amongst these challenges, clinical competency achievement is the primary goal of the resident. Surgical residents focus on technical competencies and surgical skills which come with practice. This situation can be worrisome in the feeling of incompetency along with other challenges. Residents can encounter conflicts during the training. All these challenges can be effectively managed with good EI. A surgeon with low EI and conflict resolution at the workplace is regarded as a negative person and team outcome. We measure the emotional intelligence of postgraduate residents in years one & two, their CC at the workplace and how they use EI to resolve conflicts.

## METHODS

We conducted a mixed-method sequential exploratory study using a pragmatism paradigm, inductive deductive, and sequential methodology, at the Departments of General and Orthopedic Surgery, Mayo Hospital, Lahore, from March 10, 2019 to May 28, 2020. After approval from the institutional review board of KEMU No. 557/RC/KEMU, dated Match 5^th^ 2019, the study was conducted in two phases one and two. In phase 1, the quantitative part was completed in which 100 PGR filled out the Mayor-Salovey-Caruso Emotional Intelligence Test (MSCEIT) questionnaire. In Phase-2, the qualitative part was completed in which 10-semi-structured in-depth interviews were done.

In phase 1, PGR of General Surgery and Orthopedic Surgery was approached in the work setting and email addresses were obtained. A personal invitation to fill out the online questionnaire was sent in an email. The email contained the purpose and objective of the study with a brief statement of consent to participate in the study. All participants completed online consent before they started the questionnaire. This questionnaire is comprised of one hundred and forty-one questions with a range of face tasks, picture tasks, facilitation, and sensations aspects of emotions. It evaluates the perceived emotions (face task & picture task), using emotion (facilitation & sensations), understanding emotion (changes & blends), and managing emotion (emotions management and emotional relation). It has four aspects of emotional intelligence for understanding emotions, including self-awareness, self-management, mindful of others, and rRelationship management. MSCEIT’s overall validity of measuring emotional intelligence is growing and arguably quite strong. After completing the response of one hundred PGRs, their EI score was calculated and graded. It was graded as improving emotions, consider developing, competent, skilled, and expert (0- <70, 70- <90, 90- <110, 110- <130 & 130-150 respectively).

In Phase-2, informed written consent was taken and qualitative data was obtained by completing a semi-structured interview of 10 PGRs. Amongst them, five participants with higher EI scores and five with lower EI scores were interviewed with a focus on their conflicts at the workplace and how emotional intelligence helps in resolving clinical conflict in surgery. Clarification of the topic under discussion and the format of the interview were briefed. The approximate length of the interview was told, and the confidentiality of data was also assured. All interviews were audio-recorded. The purpose of a digital recorder, its permission to use, and who will listen to the recording were thoroughly explained.

The questions were developed in the native language and were translated to English keeping the objectives of the study. In the case of female residents’, the interview was conducted in the presence of a female chairperson, and his/her views were not recorded as a part of the data collection. Transcription of the data was completed after the next day of the interview.^14^ Questions that were asked during this study were; what do you know about emotional intelligence, i.e., motivation, empathy, self-awareness, self-control, and social skills? What is the clinical conflict in your workplace? How did you solve the clinical conflict among the residents? How do your personal experience and professional knowledge help you in this regard? Do you think that it affects your personal and team performance? Do you believe that residents need training in emotional intelligence?

### Statistical analysis

Quantitative data were entered and analyzed using MS Excel. Descriptive statistics like the mean and the standard deviation were calculated for age, perceiving, using, understanding, and managing emotions and experiential and strategic scores. Frequencies and percentages were calculated for all qualitative variables like gender, year of training, and emotional intelligence grading of perceiving, using, understanding, and managing emotions. Independent sample t-test was used for the age of participants with perceiving, using, understanding, managing, experiential, area, and overall grading was used, and a p-value <0.05 was taken as significant.^15^ Qualitative data were analysed using a thematic analysis of the framework.

### Data interpretations

The transcript of the interviews was read by a second author in detail and employed the six-step approach to thematic analysis proposed by Braun and Clarke et al.^16^ as familiarisation with the data; generating initial codes; searching for themes; reviewing themes; defining and naming themes, and producing the report.

## RESULTS

Ninety-one (91%) have not improving EI, and 09 (09%) have considered developing EI. The demographic details and EI grading are given in Table-I. The overall emotional intelligence score along with other core variables of EI scores. (Table-II). The minimum and maximum scores with means and SD of interviewers PGRs with lower EI were 18.6 to 24.5 (13.1±7.4) while for the higher EI was 50.9 to 75.5 (60.8±13.1).

**Table-I:** Demographic data emotional intelligence grading of the participants.

Variable	Frequency N=100	Percentage %
***Gender of the participants***		
Male	76	76%
Female	24	24%
Age of the postgraduate resident	28.97	4.819
***Postgraduate year of training***		
Year 1	48	48%
Year 2	52	52%
***Emotional intelligence grades***		
Improving (0-<70)	91	91%
Consider developing (70-<90)	09	09%
Competent (90-<110)	00	00%
Skilled (110-<130)	00	00%
Expert (130-150)	00	00%

**Table-II:** Overall Emotional intelligence grading of the postgraduate residents.

Variable	Minimum score	Maximum score	Means ±SD	p-value
Overall Emotions	18.16	75.4	46.2±14.8	=0.577
Perceiving Emotions	1.92	31.1	17.5±6.8	=0.879
Using emotions	2.4	14.2	8.6±2.6	=0.696
Understanding emotions	3.4	21.5	11.07±4.3	=0.443
Managing emotions	16.5	3.6	9.003±3.1	=0.664
Experiential area score	4.3	43.2	26.1±9.01	=0.853
Strategic area score	9.004	38.2	20.08±7.02	=0.541

### Four themes were generated

1. emergency with sub-themes (1a) gender bias, (1b) lack of professionalism, (1c), nepotism (1d) toxic culture. 2. Elective operation theatre (2a) burn out, (2b) nepotism. 3. Outpatient department (3a) lacks professionalism. 4. Ward (4a) toxic culture, (4b) burn out (4c) lack of professionalism.

Six strategies: 1. Disgruntle when alone, 2. gender affinity, 3. deceit, 4. performing attention-attaining work, 5. interlude, 6. self-study.

### Emergency

Case allocation was one of the significant conflicts postgraduate residents faced during their duty in an emergency. They were not allotted the major procedures; had to observe the consultants doing the procedures and could not give their opinions during the diagnosis. While assisting procedures, they have gender issues of putting the third assistant. (1a) gender bias. Preferences were given to female genders by seniors, and viewpoints by both male and female participants. They felt extreme danger and threats in the workplace. The female postgraduate residents viewed being married as a blessing. “Thank God I am married” and “Female surgeons were not encouraged and supported to do the surgery”. Major procedures were allotted to the favourite postgraduate residents. Favouritism and friendship, as said by female postgraduate residents was healthy if it was in the sense of learning and competition. But if it was considered in the sense of favours based upon personal likes and dislikes, it caused frustration. (1b) lack of professionalism; Diagnosis of the patient was reported as a conflict; in an emergency, the patient might come with more than one problem. Diagnosis presented by the trainee to the team leader and senior registrar was rejected most of the time, with most viewed as a conflict of opinion and lack of personal and professional grooming at the workplace. Lack of professional and personal grooming of seniors and consultants regarding several factors was a conflict complained by all the participants. Previously, the conditions were fine, and now things are getting worse. The biggest issue is the ego of consultants.” (1c) Nepotism; There was an emotional outburst of conscious behavior and numb eyes, and it was a different experience to obtain the conflict view of the participants. Case allocation by the team leader in the emergency was not proportionate with senior. Working with disrespect, facing emotional aggression and physical push of body was very hurting. (1d) Toxic culture; Due to the grouping system, postgraduate residents were not able to learn healthy things and perceived the negative impact of seniors. Closer friends gain favours with an evident lack of ability of pre-op knowledge to juniors and the majority have a feeling of inferiority complex due to conflict of opinion and scepticism.

### Elective operation theatre

They observed that the senior registrar was learning how to become an excellent surgeon, and this culture was creating a fearful situation of training at the workplace. The curriculum was not defined with an evident lack of knowledge of achieving competencies in elective operation theatre in years one and two of training. They seek cases in different ways or are allotted to them by seniors without supervision. The system of doing elective surgery of assistant professor and above was well managed in elective operation theatre; they saw their senior registrars stay on the table with them. The engagement of the juniors with seniors in specific cases was somehow accessible with a lack of supervision. (2a) Burnout: Seniors who coded the mistakes of the juniors openly felt embarrassed and threatened by their personalities. It greatly affected their training. They needed much work, and time to resolve this conflict. It decreased their energy to work and collaborate in a team. All residents faced emotional outbursts while assisting their seniors. It splashed their concentration as an assistant ultimately blurred their learning. (2b) nepotism; If the juniors are assisting in surgery, then a small mistake by postgraduate residents was highly exaggerated by the consultant. If the case was somehow difficult, a big issue was created by the seniors.

### Outpatient department

In the outpatient department, no work was defined. Patient overload, lack of infrastructure, and routine way of seeing and admitting patients hamper the case discussion with seniors and missed clinical examinations in most of the patients. The patients were diagnosed by house officers and postgraduate residents; consultants did not pay attention to it. Time management had been a conflict. Many things were missed regarding diagnosis. Most postgraduate residents said that they learn things by personal observation. (3a) lack of professionalism; The follow-up of the cases in the outpatient department was poor, with the constant switching of the patient to a different doctor on every visit.

### Ward

According to participants, the Study curriculum was not defined in the ward settings as well; they faced difficulty in preparing for rounds. Some postgraduate residents faced politics and professional jealousy of their colleagues. Residents reported conflict that consultants asked irrelevant questions during the rounds. They felt that they were being targeted in the ward round. The difference in post-operative decisions during ward rounds carried a challenging situation for bed and ward week doctors. They took the operating surgeon on board to follow the final decision regarding patients. (4a) Toxic culture; Most of the surgeons felt embarrassed, and low esteemed during round questions asked in front of nurses and paramedics, as they did not know the answers to the questions asked by the surgeon. Favourite people got a presentation, while some doctors reported that they were not given even a single presentation during their whole study tenure of 18 months in general surgery. They had to follow the seniors, as they had no opinion. The seniors have to be followed blindly. (4b) burnout. Conflicts were seen largely regarding work and learning. Personality was damaged by the behaviour of seniors and consultants. Female postgraduate residents faced negative behaviour from the people of other departments; they did not get comfortable while working with them. The emotional mood of seniors and consultants affected all participants. I feel that I am not important and mentally tortured. (4c) lack of professionalism. During the round, the consultants made round orders differently over a single patient. The lack of communication and professional approach among the consultants was difficult to manage. Everyone tried to run their things; there was no coordination, so there were problems. The seniors have to be followed blindly.

### Conflict resolution strategies

Six conflict resolution strategies were adopted by the postgraduate residents as disgruntle when alone. Most adopted abusive behaviour at home while stating their family now understands their training-related hassle was the reason. Abusing others when alone was a peaceful feeling.

### Gender affinity

People did all sorts of things to get the attention of seniors and consultants. Specific tables were given to specific female residents’ procedures.

### Deceit

Look busy, do nothing Non-favourite Residents got short leave under the umbrella of personal or relative check-ups to stay away from work when the consultants gave short leave to their favourite residents. residents used to take the opinion of both sides in the personal and clinical conflict of managing the situation and mostly go with the management plan of a senior consultant versus a junior consultant.

### Performing attention-attaining work:

Most participants said that they had to do overwork to get noticed by the seniors and consultants. they stayed awake to get a case in the last 24 hours of an emergency, and it was very abnormal for them.

### Interlude

They also wait for their time to become seniors to get access to the same opportunities. They feel fear of the level of skill set when they become a consultant. They perceive the situation negatively observing the clinical and surgical skills of the seniors growing up in this same culture.

### Self-study

Managed to read books, supervise juniors, and take help from YouTube videos in learning, but it was not a satisfactory method for them.

## DISCUSSION

Overall emotional intelligence score in this study was very low. The majority, 91%, were improving emotions, and only 09% were considered developing emotions. The mean score of emotional intelligence in this study was 46.2±14.8. Compared to McKinley et al., 2015^17^, who measured EI using global standardized emotional intelligence, reported a score of 101.0 ± 8.1. The reason for the low score in this study was the lack of awareness, teaching, and training of emotional intelligence in postgraduate residents.

Understanding the emotion score was very low (100% were improving emotions). Residents were struggling in all four settings, including emergency, outpatient department, ward, and elective operation theatre. They were not able to understand the professional conflicts and manage accordingly. The minimum and maximum scores of understanding were 21.5 and 3.14, respectively (mean=11.078±4.328) with a Pearson correlation were -0.015 (p-value=0.443). Residents were initially struggling with the ability to understand themselves and other emotions, but the situation was improving with awareness and management.^18^ The data importance of EI has been ignored to seek an understanding of how health professionals can improve their knowledge and skills. Emotional aspects of clinical conflict reasoning are lacking in the literature. The literature is reported on nurses, but critical thinking and clinical reasoning referenced emotions are missing.^19^,^20^

It has been seen extensively that during the needs and ways to understand clinical knowledge and skills, health professionals ignored the role of emotions and the importance of emotional intelligence. It is highlighted that negligence during clinical reasoning indicated the absence of attention to PGS.^17^,^18^ In the current study, the participants responded that it was complex and challenging to deal with emotional intelligence. They said that emotional experience was vital and was of great importance while making clinical decisions. The participants in this study covered emotions to trigger, inform, and increase the process of clinical decisions.^19^-^21^

Burnout was the most common sub-theme in this study. Riaz et al.^22^ reported similar findings to our study having a high stress rate in postgraduate residents. Similarly, distraction was the coping strategy to manage the stress was comparable to this study in which residents’ adept deceit strategy to manage the burnout. Sohail et al.^23^ reported high self-awareness of emotions and low scores in managing emotions in postgraduate residents. The results of this study are similar to Sohail et al in managing emotions while understanding emotions score was low in our study. During clinical reasoning and decision-making, the involvement of emotions was confirmed. It was identified and confirmed that emotions were indispensable features of human cognition.^22^ It’s recognized that emotion and cognition processing are integrated with the brain and thereby jointly contribute to behaviour, particularly memory, attention, and decision-making.^23^ Residents with low scores felt low self-esteem and were equally struggling in managing conflicts in our society. None of the residents was aware of emotional intelligence attributes.

Residents did not report EI training. A study reported that participants step back and think about why I am responding to the situation in that way while residents did eat more, stay away from the workplace and show aggression is alarming in my study.^24^ All felt depressed in an emergency. Sharing with family and colleagues was the most relieved and satisfactory method for residents. With the help of this study, we were able to report the problems related to emotions, which we take for granted. The special behaviour of the resident’s workplace can easily be identified and addressed. The overall emotional intelligence of the residents made none of them able to identify and use emotional sense to resolve the conflicts. This study has added to the gap of overall emotional intelligence of PGR. They were on emotionally intelligent in perceiving, understanding, using and managing emotions of self and others. PGRS with higher and lowers EI score have similar conflicts and conflict resolution strategies at four different settings of workplace indicate a lack of EI. The conflict resolution strategies were consistent with social grooming and lack professional sense in PGRS.

### Limitations

This study has a limitation in that was done with people confined to year one and two trainees. There is a possibility that if this study can be further extended at the institutional or managerial level, there is a huge possibility those problems could be identified, which till now are taken for granted or overlooked.

## CONCLUSION

Emotional intelligence was lacking in all PGR. They were unable to talk about the attributes of the EI and its relevance in clinical settings. The majority have conflict in an emergency. Most reported clinical conflicts were prolonged duty hours, difficulty in presenting diagnosis, friendship culture, dealing with another speciality, and emotional aggression in assisting procedure.

The most adopted strategies were self-study, deceit, gender affinity, performing attention-attaining work, aggrieved reaction and disgruntle when alone. Gender friendship with seniors was alarming, and both sides viewed it as a culture that is an unprofessional, unethical aspect of the workplace. The development of anxiety and fear were present in all participants of the interview with irrational behaviour at the workplace and home.

### Authors Contribution:

**SFUHSG:** Conceived, designed and did statistical analysis, wrote & edited of manuscript, is responsible for integrity of research.

**RAH & AS:** Helped in manuscript writing, critically revised, statistical analysis,

**AS:** Did review and final approval of manuscript.

## References

[1] Missouridou E (2017). Secondary Posttraumatic Stress and Nurses'Emotional Responses to Patient's Trauma. J Trauma Nurs.

[2] Ilaslan E, Adıbelli D, Teskereci G, Cura SU (2023). Development of nursing students'critical thinking and clinical decision-making skills. Teach Learn Nurs.

[3] Bari A, Khan RA, Rathore AW (2016). Medical errors;causes, consequences, emotional response and resulting behavioral change. Pak J Med Sci.

[4] Afzalur Rahim M, Antonioni D, Psenicka C (2001). A structural equations model of leader power, subordinates'styles of handling conflict, and job performance. Int J Confl Manag.

[5] Taksic V The Importance of emotional intelligence (competence) in positive psychology. Poster Present First Int Posit Psychol Summit.

[6] Kozlowski D, Hutchinson M, Hurley J, Rowley J, Sutherland J (2017). The role of emotion in clinical decision making:an integrative literature review. BMC Med Educ.

[7] Ibrahim NK, Algethmi WA, Binshihon SM, Almahyawi RA, Alahmadi RF, Baabdullah MY (2017). Predictors and correlations of emotional intelligence among medical students at King Abdulaziz University, Jeddah. Pak J Med Sci.

[8] Petrides KV, Pita R, Kokkinaki F (2007). The location of trait emotional intelligence in personality factor space. Br J Psychol.

[9] Imran N, Awais Aftab M, Haider II, Farhat A (2013). Educating tomorrow's doctors:A cross sectional survey of emotional intelligence and empathy in medical students of Lahore. Pak J Med Sci.

[10] Arora S, Ashrafian H, Davis R, Athanasiou T, Darzi A, Sevdalis N (2010). Emotional intelligence in medicine:a systematic review through the context of the ACGME competencies. Med Educ.

[11] Kasi PM, Khawar T, Khan FH, Kiani JG, Khan UZ, Khan HM (2007). Studying the association between postgraduate trainees'work hours, stress and the use of maladaptive coping strategies. J Ayub Med Coll Abbottabad.

[12] Saaiq M, Khaleeq-uz-Zaman (2013). Postgraduate medical education:residents rating the quality of their training. J Coll Physicians Surg Pak.

[13] Zia S, Onali MA, Yousuf H, Masoom A, Shahab A, Amjad N (2020). Perceptions of Fellowship Trainees in Public and Private Tertiary Care Hospitals of Karachi. J Islamabad Med Dent Coll.

[14] Bernard H (2000). Social Research Methods:Qualitative and Quantitative Approaches, Sage, Thousand Oaks.

[15] Jung YM Data Analysis in Quantitative Research. Handb Res Methods Heal Soc Sci.

[16] Braun V, Clarke C (2006). Using thematic analysis in psychology. Res in psychol.

[17] McKinley SK, Petrusa ER, Fiedeldey-Van Dijk C, Mullen JT, Smink DS, Scott-Vernaglia SE (2015). A multi-institutional study of the emotional intelligence of resident physicians. Am J Surg.

[18] Simmons B (2010). Clinical reasoning:concept analysis. J Adv Nurs.

[19] Von Colln-Appling C, Giuliano D (2017). A concept analysis of critical thinking:A guide for nurse educators. Nurse Educ Today.

[20] Evidence-Based Practice for Nurses and Healthcare Professionals (Third edition) Barker Janet Linsley Paul Kane Ros Evidence-Based Practice for Nurses and Healthcare Professionals (Third edition) (2016). Nurs Stand.

[21] Anderson CJ (2003). The psychology of doing nothing:Forms of decision avoidance result from reason and emotion. Psychol Bull.

[22] Riaz Q, Ali SK, Khan MR, Alvi AR (2021). Stress and coping among surgery residents in a developing country. J Pak Med Assoc.

[23] Sohail M, Yasmin R, Ahmad A, Illyas R (2020). Emotional Intelligence of Medical and Dental Doctors with Different Clinical Experiences:A Cross Sectional Study. J Islam Int Med Coll.

[24] Alanazi SA, Shabbir M, Alshammari N, Alruwaili M, Hussain I, Ahmad F (2023). Prediction of Emotional Empathy in Intelligent Agents to Facilitate Precise Social Interaction. Appl Sci.

[25] Sadati L, Yazdani S, Heidarpoor P (2021). Surgical residents'challenges with the acquisition of surgical skills in operating rooms:A qualitative study. J Adv Med Educ Prof.

